# Effect of Incorporation of Bioactive Glass-Ceramic into Self-etch Adhesives

**DOI:** 10.3290/j.jad.b2916451

**Published:** 2022-04-13

**Authors:** Fernanda de Carvalho Panzeri Pires-de-Souza, Rafaella Tonani-Torrieri, Rocio Geng Vivanco, Carolina Noronha Ferraz de Arruda, Saulo Geraldeli, Mário Alexandre Coelho Sinhoreti, Jean-Francois Roulet

**Affiliations:** a Full Professor, Department of Dental Materials and Prosthodontics, Ribeirão Preto School of Dentistry, University of São Paulo, Ribeirão Preto, Sao Paulo, Brazil. Experimental design, performed the experiments, wrote the manuscript, perfomed statistical evaluation.; b Laboratory Technician, Department of Dental Materials and Prosthodontics, Ribeirão Preto School of Dentistry, University of São Paulo, Ribeirão Preto, Sao Paulo, Brazil. Performed the restorations.; c PhD Student, Department of Dental Materials and Prosthodontics, Ribeirão Preto School of Dentistry, University of São Paulo, Ribeirão Preto, Sao Paulo, Brazil. Wrote the manuscript.; d Researcher, Department of Dental Materials and Prosthodontics, Ribeirão Preto School of Dentistry, University of São Paulo, Ribeirão Preto, Sao Paulo, Brazil. Proofread the manuscript.; e Associate Professor, School of Dental Medicine, East Carolina University, Greenville, NC, USA. Experimental design.; f Full Professor, Department of Restorative Dentistry, Piracicaba Dental School, State University of Campinas, Piracicaba, Sao Paulo, Brazil. Contributed substantially to discussion.; g Professor, Department of Restorative Dental Sciences, University of Florida, Gainesville, FL, USA. Contributed substantially to discussion.

**Keywords:** degree of conversion, wettability, bond strength, bioactive glass-ceramic, self-etch adhesive

## Abstract

**Purpose:**

This study evaluated the effect of incorporating different concentrations of biosilicate in an experimental self-etch adhesive (SE).

**Materials and Methods:**

Biosilicate microparticles (0, 2, 5, and 10 wt%) were incorporated into the primer, and degree of conversion (DC) and wettability were tested (one-way ANOVA, Tukey’s test, p < 0.05). The two best concentrations were selected (2% and 5%) for µTBS evaluation. Sound human molars (n=20) were sectioned into quarters and randomly assigned to 4 experimental groups: 1. experimental SE + 0% biosilicate (Exp0%; negative control); 2. experimental SE + 2% biosilicate (Exp2%); 3. experimental SE + 5% biosilicate (Exp5%); 4. AdheSE (Ivoclar Vivadent, positive control). After adhesive application, Filtek Z350 (3M Oral Care) composite was built up incrementally to 5 mm. Each quarter tooth was sectioned into sticks (0.9 mm^2^) and stored in distilled water (37°C) for 24 h, 6 months, or 1 year. After storage, sticks were submitted to µTBS (0.75 mm/min). The Ca:P ratio was analyzed using scanning electron microscopy (SEM) and energy-dispersive x-ray spectroscopy (EDS). Data were analyzed using two-way ANOVA with Bonferroni’s correction, with statistical siginificance set at p < 0.05. Fracture patterns were observed under a digital microscope and adhesive interfaces with transmission electron microscopy (TEM).

**Results:**

Exp2% presented the highest DC (p < 0.05), Exp5% exhibited the lowest µTBS (p < 0.05), and adhesive failures were predominant in all groups. TEM suggested remineralized areas in Exp2% and to a lesser degree in Exp5%. Exp2% and Exp5% showed a higher Ca:P ratio after aging (p < 0.05).

**Conclusion:**

The incorporation of biosilicate microparticles can improve the properties of self-etch adhesives. It increased the DC of the experimental adhesive as well as mineral deposition. However, the adhesive properties are concentration dependent, as a higher concentration of microparticles can adversely affect the mechanical properties of an adhesive.

Over the last decade, there has been a significant increase in the use of bioactive materials due to their unique ability to interact with mineralized tissues.^17,34,36^ The bioactive glasses (BAGs) stand out among them, showing high potential in clinical and laboratory studies.^29,36,39,40^ Developed by Larry Hench in 1969,^20^ these biomaterials can form chemical bonds with bone and dental tissues. In contact with body fluids, they release calcium and phosphate ions,^8^ and form a hydroxycarbonate apatite layer (HCA) with a structure and chemical composition similar to the mineral phase of these tissues.^19^

BAGs are biocompatible materials that stimulate new tissue formation and thus have multiple clinical applications in medicine and dentistry, demonstrating excellent results in tissue engineering.^15,36^ They have been commercialized in several successful medical products in solid form (bulk and porous scaffolds), as powder (for deposition of coatings on biomedical devices or directly on mineralized tissues), and as composites (acting as fillers).^4,15^ In dentin, studies have demonstrated that bioactive glasses are effective materials for dentinal desensitization. They occlude the dentinal tubules, leading to interruption of neural activation and painful stimuli.^9,39,40^ In addition, as a result of ion release, the local pH becomes alkaline, promoting anticariogenic action and tooth remineralization.^43^

In the last few years, bioactive materials have not only been used as surface pretreatment, but also incorporated into composites.^12,16^ Hydroxyapatite has been added to adhesives^1,21,31^ to improve their physical and mechanical properties, thus increasing the durability of the hybrid layer. It enhances the bond strength of the adhesive to dentin by penetrating the dentinal tubules, decreasing polymerization shrinkage and increasing the elastic modulus of the hybrid layer.^1,31^ Unfortunately, depending on the concentration and morphology of the particles, its incorporation could bring some disadvantages, such as reduction of the dentin wettability by the adhesive resin and incomplete polymerization;^21^ since light can be reflected and absorbed by the filler particles, attenuating it. This certainly affects camphorquinone excitation and, consequently, reduces the DC of the adhesive.^21,31^

Bioactive glasses, such as Bioglass 45S5, Zn-polycarboxylate BAG, and niobophosphate bioactive glass (NbG), have also been incorporated into commercial and experimental adhesives.^3,5,36^ They reduce micropermeability along the adhesive interface by remineralization of the dental tissue, increasing the modulus of elasticity and hardness of the hybrid layer. Nonetheless, their incorporation can affect the physicochemical and mechanical properties of the adhesives. The bioactive glasses have shown ambiguous results regarding their effect on the adhesive bond strength and the degree of conversion of adhesives.^3,13^ Thus, the bioactivity of these materials and their influence on the adhesives’ properties depend on the structure, composition, and concentration of the glass.^13,36^

Biosilicate is a fully-crystallized highly bioactive glass-ceramic developed to combine the high bioactivity of bioactive glasses such as Bioglass 45S5 (NovaMin, GlaxoSmithKline; London, UK), and the good mechanical properties of some glass-ceramics.^8^ In contact with oral fluids, it promotes the formation of HCA in mineralized tissues,^8^ as well as the occlusion of open dentinal tubules.^9^ Moreover, it exhibits a wide spectrum of antimicrobial activity, being effective against anaerobic bacteria.^23^

Regarding restorative dentistry, a study performed by de Morais et al^11^ demonstrated that dentin pretreatment with biosilicate particles prior to the application of an etch-and-rinse adhesive positively influenced the bond strength. Furthermore, it did not interfere with the bonding ability of a self-etch adhesive.^11^ Some studies^10,41^ proved that its use on sound and artificial caries-affected dentin increased the bond strength of the etch-and-rinse adhesive. However, the performance of the self-etch adhesive applied on caries-affected dentin did not improve significantly.

The success of a restorative procedure depends on the bonding ability of the adhesive.^41^ Special attention should be given to the self-etch adhesives due to their limited conditioning ability.^41^ Biosilicate has not yet been incorporated into adhesives. Therefore, it is important to evaluate the effect of adding different concentrations of this bioactive glass-ceramic on the physicochemical and mechanical properties of adhesives. The incorporation should be controlled to yield satisfactory results.

Considering the absence of reports on the addition of biosilicate microparticles to self-etch adhesives and its proven benefits, which include induction of dental remineralization and anticariogenic action, the aim of this study was to evaluate the effect of the incorporation of different concentrations of biosilicate microparticles on the physicochemical and mechanical properties of an experimental self-etch adhesive and its influence on the characteristics of the hybrid layer. The null hypothesis tested was that regardless of the concentration, the incorporation would not affect the degree of conversion, dentin wettability by the adhesive resin, and the dentin bond strength of the experimental self-etch adhesive.

## Materials and Methods

After approval by the Research Ethics Committee (CAAE #53308816.0.0000.5419), 36 sound human third molars were selected and disinfected with 0.1% thymol. Two adhesives were used (Table 1): An experimental two-step self-etch adhesive and a commercial one (AdheSE, Ivoclar Vivadent, Schaan, Liechtenstein). A pilot study was performed to select the two minimal particle concentrations to be added to the adhesive without negative effects on the degree of conversion and wettability of the material. Thus, 0 (control), 2, 5, and 10 wt% of biosilicate microparticles (Vitrovita; São Carlos, SP, Brazil; Table 1) were incorporated into the primer of the experimental adhesive.

**Table 1:** Materials used

Materials	Composition (% by weight)		Manufacturer
Experimental self-etch adhesive	Primer	GDMA-P (15)	–
		TEG-DMA (15)	
		HEMA (30)	
		Ethanol (20)	
		Water (20)	
	Bonding agent	Bis-GMA (50)	
		Bis-EMA (10)	
		TEG-DMA (20)	
		UDMA (10)	
		CQ (0.5)	
		DMAEMA (0.5)	
AdheSE	Primer: acrylic ether phosphonic acid, bisacrylamide, water, CQ, stabilizers		Ivoclar Vivadent; Schaan, Liechtenstein
	Bonding agent: bis-GMA, GDMA, HEMA, fumed silica, CQ, tertiary amine, stabilizers		
Biosilicate	Fully-crystallized highly bioactive glass-ceramic (P_2_O_5_-Na_2_O-CaO-SiO_2_), mean size = 4 µm		Vitrovita; São Carlos, SP, Brazil
Filtek Z350 XT	Bis-GMA, bis-EMA, UDMA, small quantities of TEG-DMA, non-agglomerated 20-nm nanoparticles of silica and nanoagglomerates formed of zirconium/silica particles ranging from 0.6 to 1.4 µm		3M Oral Care; St Paul, MN, EUA

GDMA: glycerol-dimethacrylate; bis-GMA: bisphenol A-diglycidyl dimethacrylate; bis-EMA: bisphenol A-polyethylene glycol diether dimethacrylate; UDMA: urethane dimethacrylate; TEG-DMA: triethylene glycol dimethacrylate; HEMA: 2-hydroxyethyl methacrylate; DMA-EMA:- Dimethylaminoethyl methacrylate; CQ: camphorquinone.

### Degree of Conversion

The degree of conversion (DC) was determined by Fourier transform infrared spectrometer (FTIR, Nicolet 380 spectrometer, Thermo Scientific; Waltham, MA, USA) equipped with an attenuated total reflectance (ATR) device, comparing the ratio between a non-polymerized and a polymerized drop of adhesive. For this, one drop of each primer and one drop of the bonding agent was dispensed directly onto the surface of the ATR diamond crystal, mixed, and measured as a non-polymerized sample. The polymerized samples were prepared in the same manner as the non-polymerized ones, except that the drops were mixed on a microscope slide and protected with a plastic film. After solvent evaporation, a second slide was used to compress the mixture and produce 0.3-mm-thick samples (n = 3) which were fully polymerized (Flash Lite 1401, Discus Dental; Culver City, CA, USA, 460-480 nm, 1100 mW/cm^2^) for 10 s. After 24 h of storage in the absence of light, readings were performed. The absorption spectrum of each sample was measured at a resolution of 1 cm^-1^. The peak heights for the aliphatic (C–C; at 1637 cm^-1^) and aromatic (C=C; at 1608 cm^-1^) carbon double bonds were recorded and used for calculation of the degree of conversion:

DC = 1- [polymerized (aliphatic/aromatic)] /

[non-polymerized (aliphatic/aromatic)] x 100.

Means were calculated for each adhesive, analyzed with the Kruskal-Wallis test, and compared using Dunn’s test (p < 0.05).

### Wettability

For the wettability analysis, the contact angle (CA) was measured using the sessile drop method. Five sound human third molars were selected and sectioned (Isomet 1000, Buehler, Lake Bluff, IL, USA) into dentin slices (2 mm thick). Dentin surfaces were polished with 600-grit SiC abrasive papers to create a uniform smear layer. Then, the slices were randomly separated into groups (n = 3), according to the adhesive tested (commercial and experimental with different biosilicate particle concentrations). Prior to applying the bonding agent, the self-etch primer was actively applied on the dentin surface for 15 s, followed by gentle air dispersion. After that, 5 µl of the bonding agent was dispensed onto the dentin. A waiting time of 1 s was standardized to stabilize the drop. The wettability was analyzed only after the adhesive application. Photographs of the angle between the dentin surface and the tangent of the drop were obtained with an automatic goniometer (CAM200, KSV Instruments; Helsinki, Finland). Then, the CA was calculated using ImageJ software.

After the analysis of the results (one-way ANOVA, Tukey’s test, p < 0.05) from the pilot study, the experimental adhesives with 2% and 5% of biosilicate microparticles showed the best results of DC and CA. Thus, they were selected to evaluate the bond strength to dentin.

### Dentin Bond Strength

Twenty sound human third molars were selected and decoronated with a diamond saw (Isomet 1000, Buehler; Lake Bluff, IL, USA). Then, the crowns were sectioned longitudinally into four quarters (mesio-vestibular, mesio-lingual, disto-vestibular, disto-lingual). The occlusal surfaces were flattened with 220-grit SiC abrasive papers to expose the dentin and polished with 600-grit SiC abrasive papers to create a standard smear layer.^18^ Each quarter from the same tooth received a different treatment (n = 20): Exp0% (negative control), Exp2%, Exp5%, and the commercial self-etch adhesive (AdheSE, Ivoclar Vivadent, positive control). For that, the self-etch primer was actively applied on the dentin surface for 15 s, followed by gentle air dispersion. Then, the bonding agent was actively applied to the dentin using a microbrush and polymerized (Flash Lite 1401, Discus Dental) for 10 s. After adhesive application, the samples were restored incrementally to create a 5-mm-thick composite build-up (Filtek Z350XT, 3M ESPE Dental Products, St Paul, MN, USA), followed by light activation for 20 s (Flash Lite 1401, Discus Dental) according to the manufacturer’s instructions. The samples were immediately sectioned according to the non-trimming technique^11^ using a cutting machine (Isomet 1000, Buehler; Lake Bluff, IL, USA), obtaining sticks of 0.9 mm x 0.9 mm. Three intact sticks were obtained per quarter, which were randomly separated to be stored in distilled water at 37°C for 24 h, 6 months or 1 year, thus yielding 1 stick per storage time and quarter. After the storage periods, they were submitted to the microtensile bond strength (µTBS) test (OM 100, Odeme Dental Research; Luzerna, SC, Brazil) at a crosshead speed of 0.75 mm/min until failure. According to the Shapiro-Wilk normality test, the data distribution was not normal. Hence, the results were analyzed with the Kruskal-Wallis test and the means compared using Dunn’s test (p < 0.05). Pre-test failures were included in the mean.

### Interfacial Fracture Pattern Analysis

Fracture patterns were observed under a digital microscope (VH-M100, Keyence; SP, Brazil, 10X magnification) and classified as adhesive, cohesive (in dentin or in resin composite), or mixed fractures.

### TEM Analysis of the Hybrid Layer

For morphological characterization of the adhesive interface, eight sound human third molars were sectioned into quarters, separated into groups, treated, and restored, as described for the bond strength test. After that, specimens of 2 mm x 2 mm were obtained (Isomet 1000, Buehler) with the adhesive interface in the middle. The specimens were fixed^38^ and then embedded in epoxy resin in appropriate molds. After curing, they were cut into 90-nm-thick slices using an ultramicrotome (EM-UC7, Leica; Vienna, Austria) with a diamond knife and analyzed using TEM (JEM-1010, JEOL; Tokyo, Japan).

### Calcium:Phosphate (Ca:P) Ratio

Semi-quantitative analysis (%wt) was conducted for each group to determine the calcium (Ca) and phosphorus (P) concentration on the hybrid layer. Three representative debonded specimens from each group were analyzed by energy dispersive x-ray spectrometry (EDS) with automatic chain. A 50-mm beam-diameter apparatus was attached to the SEM (EDX, JSM-5600LV, JEOL; Tokyo, Japan) to identify elemental composition of the treated dentin surface in backscatter mode. Ten random areas on the hybrid layer were selected with a resolution of 7 µm and each spectrum was acquired for 100 s (15 kV and 20 mm working distance). Data were calculated using the Ca:P ratio as the emitted x-ray parameters. Three measurements were obtained from each specimen and means were calculated for the statistical analysis (two-way ANOVA, Bonferroni adjustment, p < 0.05).

## Results

### Degree of Conversion (DC)

**Fig 1:**
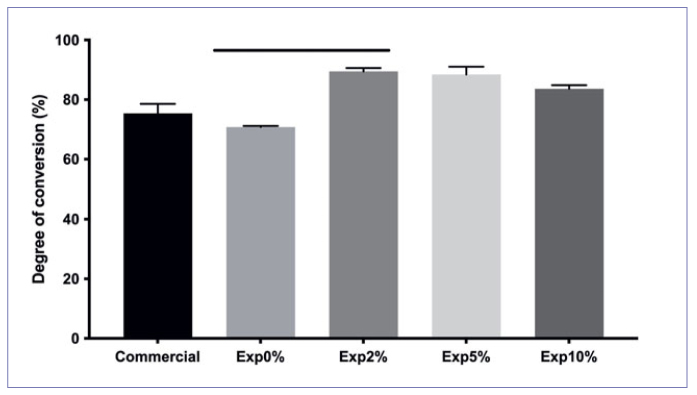
DC (%) for the experimental groups (Kruskal-Wallis, Dunn’s test, p < 0.05). The two columns under the horizontal bar are significantly different (p < 0.05).

The DC means are shown in Fig 1. Similar DCs were observed for the tested adhesives (p > 0.05), except for Exp0% vs Exp2% (p < 0.05). No other significant differences were found. Exp2% and Exp5% presented the highest DC, but this was not statistically significant (p > 0.05).

### Wettability

**Fig 2:**
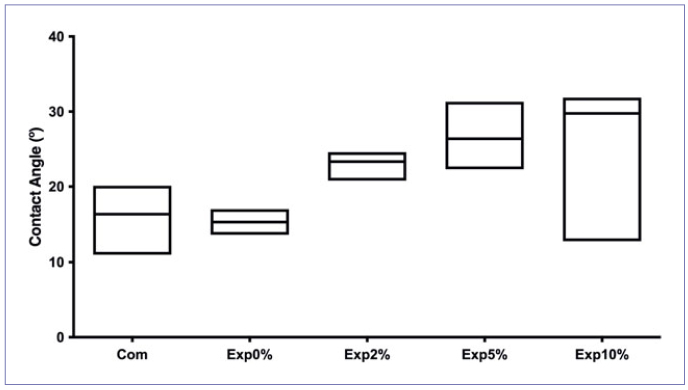
Contact angle (degrees) in each experimental group.

Corresponding contact angles are given in Fig 2. Regardless of the biosilicate concentration, all the adhesives demonstrated similar contact angles (p > 0.05). Nevertheless, Exp10% showed great variability.

### Dentin Bond Strength

The bond strength means and standard deviations are presented in Table 2. Regardless of the storage period, Exp5% demonstrated the lowest bond strength. No significant differences among the storage periods were observed for any of the tested adhesives (p > 0.05).

**Table 2:** Means and standard deviations of microtensile bond strength (MPa) for all experimental groups

	Commercial	Exp0%	Exp2%	Exp5%
24 h	32.4 (15.8) aA	36.2 (14.3) aA	28.7 (11.0) aA	15.5 (9.0) bA
6 months	26.0 (10.4) bA	40.1 (15.0) aA	31.1 (14.6) abA	14.3 (6.8) bA
1 year	28.11 (13.05) abA	29.5 (15.0) abA	34.6 (12.24) aA	13.9 (14.39) bA

Different letters (lowercase in rows and uppercase in columns) indicate statistically significant differences (Kruskal-Wallis, Dunn, p < 0.05).

### Interfacial Fracture Pattern Analysis

**Fig 3:**
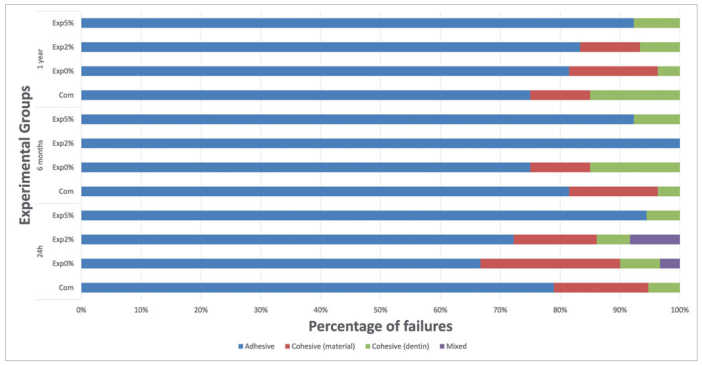
Fracture patterns of the tested groups.

Figure 3 demonstrates that the most prevalent fracture pattern for all groups was adhesive. Cohesive fractures in dentin occurred in all groups, except for Exp2% after 6 months. Cohesive fractures in resin composite were also found in all the groups, except for Exp5%, irrespective of the storage periods, and for Exp2% after 6 months. On the other hand, after 24 h, Exp0% (negative control) and Exp2% showed some mixed fractures.

### Hybrid Layer Analysis

**Fig 4:**
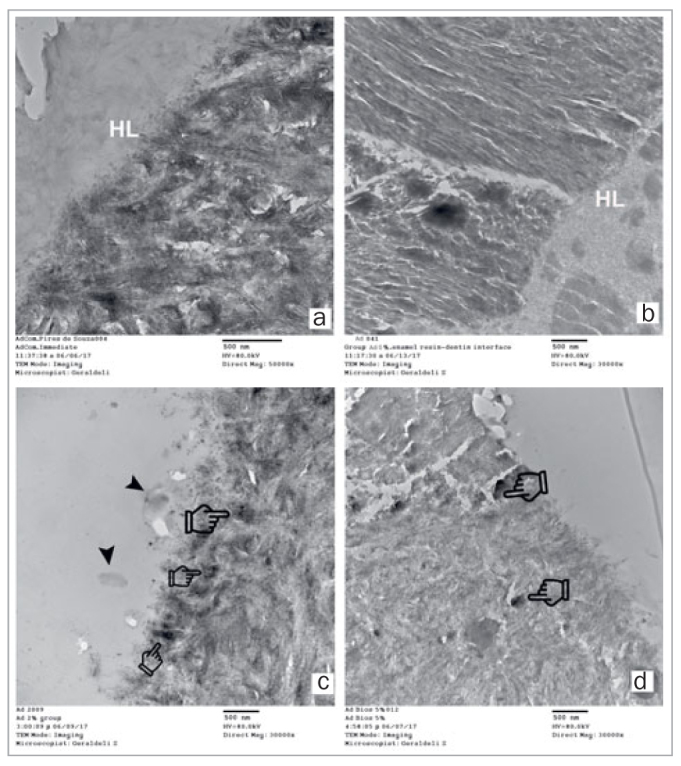
Representative TEM images of the hybrid layer created on dentin, aged for 1 year, with a) Commercial; b) Exp0%; c) Exp2%; and d) Exp5% adhesives. HL: hybrid layer; arrow: biosilicate microparticles; pointer: remineralized area.

Representative TEM images of the hybrid layer after 1-year storage are presented in Fig 4. In Figs 4c and 4d, biosilicate microparticles were seen within the hybrid layer in contact with the collagen fibrils; the hybrid layer was thicker with Exp2%.

### Ca:P Ratio

Table 3 shows the comparison of the Ca:P ratio between the tested groups. There were statistically significant differences (p < 0.001) between the storage periods in all groups, except for Exp0% (p > 0.05).

**Table 3:** Means and standard deviations of the Ca:P ratio for all experimental groups

	Commercial	Exp0%	Exp2%	Exp5%
24 h	2.20 (0.03) bA	2.47 (0.04) aA	2.16 (0.01) bA	2.16 (0.01) bA
6 months	1.98 (0.02) cB	2.49 (0.03) aA	2.40 (0.03) abB	2.31 (0.04) bB
1 year	2.58 (0.04) aC	2.49 (0.10) aA	2.38 (0.04) bB	2.27 (0.06) cB

Different letters (lowercase in rows and uppercase in columns) indicate statistically significant differences (p < 0.05).

## Discussion

The aim of this study was to evaluate the influence of different biosilicate microparticle concentrations incorporated into an experimental two-step self-etch adhesive on the DC, wettability, and bond strength to dentin, using a commercial self-etch adhesive as control. The null hypothesis was that regardless of the concentration, the addition of biosilicate microparticles would not affect the DC, the dentin wettability by the adhesive resin, or the dentin bond strength of the experimental self-etch adhesives. Considering the results, the null hypothesis was rejected. Exp2% resulted in a higher DC than did Exp0%. The bond strength of Exp5% decreased significantly and a non-significant decrease was also noted for Exp2% after 24 h and 6 months. Furthermore, the Ca:P ratio decreased significantly when biosilicate particles were incorporated into the experimental adhesive (except for Exp2% after 6 months). However, they had no effect on the dentin wettability.

According to Fig 4, all the tested adhesives which were applied in self-etch mode formed uniform hybrid layers integrated within the dentin. Self-etch adhesives demineralize the dentin only superficially, leaving residual hydroxyapatite crystals attached to the collagen fibrils available for possible chemical interaction, since there is no rinsing step required. Furthermore, there is little water in superficial dentin,^37^ and there is a high content of bis-GMA molecules that act as cross-linking agents, yielded desirable mechanical properties.^30^ Specifically, the commercial adhesive used in the present study contains acrylic ether phosphonic acid as the hydrolytically stable monomer that chemically binds to the calcium ions of the hydroxyapatite. Moreover, the methacrylate group of these monomers could attach to hydrolytically stable bisacrylamide cross-linkers.^32^ These reasons may explain the good bond strength results obtained for the commercial and Exp0% adhesives.

Both the commercial and Exp0% adhesives also presented a high concentration of calcium and phosphate ions in the hybrid layer after 1 year of aging.^32^ Acidic monomers in self-etch primers remove calcium and phosphate ions from hydroxyapatite, but since there is no rinsing step, they remain dispersed within the dentin surface as an amorphus precipitate. In addition, acidic monomers containing negatively charged phosphonate or phosphate groups can attract positively charged calcium ions that accumulate on the surface. The surface thereby acquires a positive charge and attracts more negatively charged phosphate ions, leading to an over-saturated surface that would explain our findings.^14,32^

Changes in the contact angles are attributable to the chemical and nanostructural characteristics of the material.^7^ Da Costa Lima et al^2^ found that 10% Bioglass 45S5 improved the wettability of a resin-based cement, but a higher concentration resulted in contact angles similar to those obtained in the control group. Similar results were found in our study with the addition of 2% and 5% biosilicate particles. However, 10% biosilicate particles altered the wettability of the experimental adhesive (Fig 2).

To ensure intimate contact of the adhesive with the tooth substrate, it is important to obtain reduced contact angles with low standard deviations, which was not observed in the Exp10% group.^25^ Furthermore, contact angles <90 degrees correspond to hydrophilic materials that better interact with the tissues.^22^ For this reason, Exp10% was not selected to evaluate the bond strength to dentin.

On the other hand, the acidic monomers of self-etch primers are neutralized when they contact calcium and phosphate ions. If no neutralization occurs, the acidic monomers could protonate the amine of the photo-initiator system present in the bonding agent, retarding the polymerization reaction and negatively affecting the overall DC.^24^ In this in vitro study, the primer and the bonding agent were dispensed directly onto the ATR diamond crystal (inert substrate without buffering); thus, Exp0% showed a low DC (p < 0.05). Conversely, the experimental adhesive with biosilicate microparticles demonstrated a better DC (Fig 1). These results agree with those of the study by Bauer et al,^3^ where the incorporation of niobium-phosphate bioactive glass (NPG) increased the DC of an etch-and-rinse adhesive. In the present study, the biosilicate probably increased the local pH and rapidly neutralized the acidic monomers.^42^ Thus, more co-initiator (tertiary amine) could survive the harshly acidic environment and improve polymerization.^43^

In another study,^6^ the addition of up to 20% bioactive glass particles (Bioglass 45S5 and NbG) did not reduce or improve the DC of an experimental self-etch adhesive. The morphology and concentration of the particles have a significant influence on the DC,^3,27,28^ as observed in the present study and in the study by Par et al.^28^ It is speculated that a high concentration of particles inhibits the polymerization by surface oxides on the BAG particles.

Little is known about ion release from biomaterials incorporated into polymers and their influence on the bond strength to dentin. The incorporation of biosilicate microparticles into an experimental self-etch adhesive aimed to increase the release and precipitation of ions and enhance the formation of hydroxyapatite, as well as improve its mechanical properties. In the present study, the Ca:P ratio analysis revealed thtat calcium and phosphate ions were released from the experimental adhesive containing biosilicate microparticles, especially after aging, demonstrating possible remineralization (Figs 4C and 4D) due to partial dissolution of the microparticles over time. Given that the mean size of the particle was 4.0 µm, the specific surface area of the particle was 4.5 x 10^5^ µm, which allows fast release of ions.

However, depending on the biosilicate particle concentration, they could decrease the mechanical strength of the adhesive^13^ and/or the etching capacity of the primer. The amount of added particles can affect the viscosity of the adhesive, hindering its penetration into the dentinal tubules. Moreover, the high concentration of ions can alter the pH and consequently the etching process,^13^ which would explain the low bond strength obtained by Exp5% and the undissolved particles found within the hybrid layer.

In contrast, Exp2% and the commercial adhesive presented similar bond strengths, which is in agreement with the results obtained by Carvalho et al.^6^ The incorporation of BAGs into an experimental self-etch adhesive resulted in bond strengths similar to those of the commercial adhesive. What is more, it presented higher bond strength than Exp5% after 1 year of aging, probably due to its lower viscosity, which facilitates handling and allows the penetration of adhesives into the dentinal tubules, thus increasing the bond strength.^13,26^

On the other hand, adhesives containing bioactive particles have demonstrated stable bond strength over time. This was also found in our study. Aging did not negatively affect the bond strength of any of the tested groups, probably due to the presence of hydrolytically stable monomers^32^ and the alkaline pH achieved with the biosilicate, which inhibits the action of endogenous proteases.^6,26^ Moreover, mineral deposition induced by this bioactive glass-ceramic can reduce the nano-infiltration after 1 year and might have mineralized and hardened the endogenous proteases, preventing hydrolytic and enzymatic degradation of the adhesive interface.^6,26^

Finally, the most prevalent fracture pattern for all groups was adhesive. Some cohesive fractures were also found, least of all with Exp5%, which is in line with the low bond strength obtained with this group. Cohesive fractures indicate that the hybrid layer remains intact.^35^ Thus, the concentration of the particles probably compromises dentin bonding, as observed by Oltramare et al,^26^ where the incidence of adhesive fractures and the number of pre-test failures increased as the concentration increased.

Further analysis is still needed to prove the interaction and benefits of biosilicate microparticles in the hybrid layer, as well as their influence on the physicochemical and mechanical properties of other types of adhesives, for example, those used in etch-and-rinse mode.

## Conclusion

The incorporation of biosilicate microparticles can improve the properties of self-etch adhesives. 2% biosilicate particles increased both the DC of the experimental adhesive and the mineral deposition. It had no effect on dentin wettability. However, the adhesive properties are concentration dependent, as a higher concentration of microparticles can adversely affect its mechanical properties. When 5% biosilicate particles were added, the bond strength decreased.

## Clinical relevance:

The incorporation of biosilicate microparticles into self-etch adhesives may promote the formation of hydroxycarbonate apatite in dentin, increasing the longevity of the restorations.
